# Supplementary material for: Comparing Factors Related to Any Conventional Cigarette Smokers, Exclusive New Alternative Product Users, and Non-Users among Japanese Youth: A Nationwide Survey
Source: Int J Environ Res Public Health. 2020 Apr 30;17(9):3128. doi: 10.3390/ijerph17093128 (PMC7246444; doi:10.3390/ijerph17093128)
Supplement: Supplementary file 1 [file ijerph-17-03128-s001.pdf]

Supplementary file 1.

Student Survey of Tobacco, e-Cigarette, and Heated Tobacco Product Use

Please circle one answer for each question:

1. Demographic data

(1) Are you male or female?

Male Female

(2) What is your school grade?

Grade 1 Grade 2 Grade 3

(3) How many years old are you?

12 13 14 15 16 17 18 19 or older

2. Use of cigarettes, e-cigarettes, and HnB

(1) Have you ever smoked a combustible cigarette including even a single puff? A combustible cigarette made from rolled paper and tobacco and smoked with fire.

No, I have not Yes, I have

(2) How many days have you smoked combustible cigarettes in the previous 30 days?

0 days 1–2 days 3–5 days 6-9 days 10–19 days 20–29 days every day

(3) Have you ever used an electronic cigarette including even a single puff? Electronic cigarettes include フレヴォ(FLEVO), エミリ (EMILI), ビタフル（VITAFUL), ビタシグ（VITASIG).

No, I have not Yes, I have

(4) How many days have you used electronic cigarettes in the previous 30 days?

0 day 1–2 days 3–5 days 6–9 days 10–19 days 20–29 days every day

(5) Have you ever used a Heat-not-Burn tobacco including even a single puff? HnB tobacco was explained by using product names to avoid any confusion; for example, heat-not-burn tobacco includes アイコス（IQOS), プルームテック (Ploom Tech), グロー (glo).

No, I have not Yes, I have

(6) How many days have you used Heat-not-Burn tobacco in the previous 30 days?

0 days 1–2 days 3–5 days 6–9 days 10–19 days 20–29 days every day

3. Environment and behavior of smoking

(1) In the past 7 days, how frequently did someone smoke near you at home?

0 days 1–2 days 3–4 days 5–6 days 7 days

(2) In the past 7 days, how frequently did someone smoke close to you outside home?

0 days 1–2 days 3–4 days 5–6 days 7 days

(3) Do you think smoking is harmful to your body?

I do not think that smoking is harmful

I think that smoking might be a little harmful but not much

I think that smoking is harmful

I am not sure

(4) Do you think that smoke from other people's cigarettes is harmful to you?

I do not think it is harmful

I think it is probably not harmful

I think it probably is harmful

I think it is harmful

4. Alcohol use

(1) How many days did you drink alcohol in the previous 30 days?

0 days 1–2 days 3–5 days 6–9 days 10–19 days 20–29 days every day

(2) How often do you drink a lot when you drink alcohol? "A lot" means "5 or more" in an ordinary can (350 ml, if it is a beer and sweet cider)?

I do not drink alcohol less than once in a year several times in a year

once or twice in a month once or twice in a week

more than three times in in a week

5. Lifestyle behaviors

(1) Do you have a breakfast every day?

I do almost every day I do sometimes I seldom do

(2) Do you participate in club activities?

I participate actively I participate but not actively I do not participate

6. School life

(1) What do you plan for your future course? Please select one the answer that is closest to your current feelings.

vocational school junior college college postgraduate school

taking a job after graduating the current school not decided yet

Supplementary file 2. The risk of exclusive alternative product use compared with exclusive cigarette use among any product users (*n* = 757).

Variables	OR	95% CI			*p*-Value
Sex					
Female	1.01	0.71	to	1.43	0.97
Male (reference)	1.00				
School grade *	0.96	0.92	to	0.99	0.02
Understand that smoking is harmful					
Yes	1.00	0.71	to	1.42	0.98
No (reference)	1.00				
Having breakfast every day					
Everyday	0.78	0.56	to	1.08	0.14
Sometimes, seldom (reference)	1.00				
Participating in club activities					
Yes	1.85	1.34	to	2.57	< 0.01
No (reference)	1.00				
Intending to pursue higher education					
College or postgraduate school	1.13	0.79	to	1.60	0.51
Others (reference)	1.00				
Second-hand smoking at home					
At least once in the preceding 7 days	1.04	0.75	to	1.42	0.83
None in the preceding 7 days (reference)	1.00				
Currently drinking alcohol					
At least once in the previous month	0.36	0.26	to	0.49	<0.01
None in the previous month (reference)	1.00				

* This variable was modelled as a continuous variable. Abbreviations: OR = odds ratio, 95%CI = 95% confidence interval
